# Effect of Low‐To‐Moderate Exogenous Carbohydrate Supplementation on Time to Exhaustion During Constant Load Intense Cycling in Healthy Individuals. A Double‐Blind, Randomised and Placebo‐Controlled Crossover Trial

**DOI:** 10.1002/ejsc.12326

**Published:** 2025-06-14

**Authors:** Janis Schierbauer, Oscar Altenberg, Thomas Voit, Paul Zimmermann, Othmar Moser

**Affiliations:** ^1^ Division of Exercise Physiology and Metabolism Bayreuth Centre of Sport Science University of Bayreuth Bayreuth Germany; ^2^ Interdisciplinary Metabolic Medicine Trials Unit Department of Internal Medicine Division of Endocrinology and Diabetology Medical University of Graz Graz Austria; ^3^ Exercise Physiology, Training & Training Therapy Research Group, Institute of Human Movement Science, Sport and Health University of Graz Graz Austria

## Abstract

Exogenous carbohydrate (CHO) supplementation during exercise is an efficient strategy to enhance endurance performance. However, the effects of low‐to‐moderate amounts of exogenous CHO on time to exhaustion (TTE) have not been fully investigated. Nineteen healthy moderately active participants (26.2 ± 1.7 years and BMI: 22.4 ± 2.3 kg·m^−2^) performed four TTE tests at the second lactate turn point (LTP2) in a double‐blind, randomised and placebo‐controlled setting consuming either 20, 40 and 60 g·L^−1^·h^−1^ of a glucose and fructose combination (CHO_20,_ CHO_40_ and CHO_60_) or a placebo (PLA). Glucose [Glu^−^] and lactate concentrations [La^−^] as well as ventilatory, heart rate (HR) and rating of perceived exertion (RPE) values were recorded during the tests. There were no statistically significant differences in TTE (PLA: 32.5 ± 9.6 min, CHO_20_: 35.9 ± 14.5 min, CHO_40_: 35.1 ± 12.9 min, CHO_60_: 38.0 ± 17.5 min and *p* = 0.11). Within each trial arm, no statistically significant differences were found between pre‐ and post‐exercise [Glu^−^] (*p* > 0.05). Post‐exercise [Glu^−^] for CHO_60_ were significantly higher when compared to PLA (*p* = 0.03). Lastly, mean ventilatory parameters as well as HR and RPE during exercise showed no statistically significant differences between trial arms (*p* > 0.05). Low‐to‐moderate amounts of exogenous CHO do not increase TTE in healthy moderately active individuals when compared to a placebo. Therefore, when exercising for less than 60 min at high intensities, exogenous CHO supplementation is not necessary if a CHO rich diet is followed prior to exercise.

**Trial Registration:**

DRKS‐ID: DRKS00030531

**Summary**

- Low‐to‐moderate exogenous carbohydrate ingestion does not enhance time to exhaustion during cycling at the LTP2 in healthy young adults with adequate pre‐exercise glycogen stores.
- Blood glucose concentrations remained stable and above hypoglycemic levels across trials, indicating it is not a primary factor in fatigue at this intensity.
- The study’s double‐blind, randomized, placebo‐controlled crossover design ensures high methodological rigor, addressing limitations in prior research on carbohydrate dosing and performance.

## 1 Introduction

Physical performance is intricately linked to the availability of energy substrates to produce adenosine triphosphate. Especially during endurance exercise, carbohydrates (CHO) play a pivotal role in sustaining prolonged efforts demands and delaying fatigue. CHO supplementation has long been established as an efficient strategy to improve endurance performance, primarily by delaying the depletion of glycogen stores and maintaining blood glucose levels (Bourdas et al. 2021; Noakes 2022).

It is usually recommended to ingest up to 60 g·h^−1^ of rapidly oxidised CHO, whereas higher amounts as much as 90 g·h^−1^ are typically not advised for athletes, as it was shown that more CHO do not offer additional performance benefits (Podlogar and Wallis 2022; Stellingwerff and Cox 2014). However, these recommendations are usually expressed for lower intensity exercise with durations longer than 60 min, mostly because it was assumed that these were nonglycogen limiting conditions, therefore exogenous CHO supplementation may not yield a positive effect (Stellingwerff and Cox 2014). This is why, if anything, it was postulated to commence short‐duration exercise with adequate muscle glycogen stores whereas during exercise itself, smaller quantities (< 60 g·h^−1^) are advised.

Normally, shorter exercise durations are associated with higher more severe intensities, that is, intensities above critical power (CP) or at the second lactate turn point (LTP2). In contrast to what was previously recommended, numerous studies have found that exogenous CHO supplementation and (or) oral mouth rinsing or exposure can improve exercise performance of tasks less than 60 min (Anantaraman et al. 1995; Below et al. 1995; El‐Sayed et al. 1997; Carter et al. 2004; Nicholas et al. 1995). Here, the amounts of CHO administered were between 25 and 130 g·h^−1^; however, the efficacy of low amounts of CHO, particularly within the range of 20–60 g·h^−1^, has not yet been comprehensively investigated, especially when exercising at higher intensities. Moreover, most of the previous studies have investigated the ergogenic effects of CHO supplementation on time trial (TT) performance, where it is well established that participants pacing during a TT is intricately linked to their individual ratings of perceived exertion (RPE) which, in turn, is dictating performance outcomes (Stellingwerff and Cox 2014; Eston 2012).

For instance, a study by Powers et al. showed that when trained cyclists performed a constant load exercise to fatigue, participants worked longer with a glucose polymer drink with 7.0 g·100 mL^−1^ compared to a nonelectrolyte placebo; however, this difference was not statistically significant (Powers et al. 1990). The amount of total CHO consumed in this study equals ∼44 g (∼60 g·h^−1^). In contrast, a study by Fielding et al. demonstrated that at very high exercise intensities (100% V̇O_2max_) until exhaustion that followed a 4 h cycling bout, participants performed significantly longer with previously administered low dose CHO supplementation (10.75 g every 30 min) compared to a control trial (Fielding et al. 1985). Although CHO feeding frequency and dosage seems to have a substantial effect on exercise performance, it remains uncertain whether low‐to‐moderate CHO supplementation offers a performance enhancing benefit, especially with regard to the lower end of CHO amounts and given that it is usually believed that a minimum of 20 g·h^−1^ of CHO is required to observe a performance benefit (Fielding et al. 1985; Jeukendrup 2004).

Intriguingly, there has been, to the best of our knowledge, only one publication using a time to exhaustion (TTE) approach, investigating equivalent power outputs between trial visits (Fares and Kayser 2011). This would allow to determine whether lower RPE would be found after the consumption of CHO when compared to a placebo. The rationale behind exploring lower CHO doses is grounded in the notion of individual variability in CHO metabolism. Although some athletes may derive optimal benefits from higher CHO intakes, others may experience similar improvements with smaller amounts (Jeukendrup and Moseley 2010), potentially reducing the risk of gastrointestinal distress, which is usually associated with larger doses (Pfeiffer et al. 2012). Therefore, understanding the performance implications of smaller, yet practical, CHO supplementation levels is crucial for tailoring nutritional recommendations to individual needs and optimising exercise outcomes. Therefore, the aim of this double‐blind, randomised and placebo‐controlled crossover trial was to investigate the effects of lesser amounts of orally administered liquid CHO (20, 40 and 60 g·h^−1^ of glucose and fructose) during constant load cycling exercise at the LTP2 on TTE in healthy moderately active individuals.

## 2 Methods

For Ethics Approval and Registration, please see title page.

### 2.1 Eligibility Criteria and Assessment of Eligibility

Eligibility criteria included male or female individuals between 18 and 35 years with a body mass index (BMI) of 18.0–29.9 kg·m^−2^. Individuals were excluded if they were simultaneously enroled in a different study, received any kind of medicinal product or had a blood pressure outside of the range of 90–150 mm Hg for systolic and 50–95 mm Hg for diastolic after resting for five minutes in a supine position. Participants were excluded if they suffered from any kind of metabolic disease, including renal or thyroid, or had a history of multiple and/or severe allergies to any trial‐related products. Inclusion and exclusion criteria were assessed by the same investigator at the screening visit prior to the start of the study.

### 2.2 Study Design

This was a single centre, double‐blind, randomised and placebo‐controlled crossover trial investigating the impact of different amounts of exogenous administered liquid carbohydrates on the time to exhaustion during constant load cycling at the second lactate turn point (LTP2) in healthy moderately active individuals. It consisted of one screening visit (V1) and four trial‐related visits (V2–5). All visits were conducted in conformity with the local COVID‐19 guidelines, which included the screening of any COVID‐19 related symptoms prior to each of the study visits. If, before any visit, a participant felt uncomfortable or deemed sick by the study team, the participant was sent home and the visit was rescheduled.

#### 2.2.1 Screening Visit

At the screening visit, participants were informed about all study‐related procedures, given instructions for the study and provided informed consent. After inclusion in the study, participants were assigned to ascending numbers and then allocated to the order in which the trial visits were conducted following a cross‐over randomised fashion using the software Research Randomizer (1:1:1:1) (Urbaniak et al.). Afterwards, participants were examined for their general health status via a physical examination including the measurement of blood pressure and a 12‐lead electrocardiogram (ECG) recording after resting for five minutes in a supine position (Amedtec ECGpro, Cardiopart 12, Straessle and Co. Medizintechnik GmbH, Albstadt, Germany). Subsequently, body composition was analysed in duplicate using a bioelectrical impedance analysis (InBody 720, InBody, Seoul, South Korea). Participants were then asked about the types of intensity of physical activity and sitting time as part of their daily lives using the International Physical Activity Questionnaire (IPAQ‐SF) (Craig et al. 2003). A venous blood sample (6 mL ethylenediaminetetraacetic acid, EDTA) was drawn from the antecubital vein for a full blood count.

To determine the individual exercise intensity for the constant load cycling sessions until exhaustion, a cardio‐pulmonary exercise test (CPX) was conducted on a mechanically remote‐controlled cycle ergometer (Excalibur Sport, Lode, Groningen, Netherlands) using a standardised three‐minute warm up at 20 W followed by 1 min incremental steps until exhaustion, that is, 20 W for males and 15 W for females.

Ventilation (V̇E), tidal volume (V̇T), respiratory rate, respiratory exchange ratio (RER), oxygen uptake (V̇O_2_) and carbon dioxide output (V̇CO_2_) were assessed via a high‐resolution portable spirometry system with breath‐by‐breath technology (Metalyzer 3B, Cortex Biophysik GmbH, Leipzig, Germany).

Additionally, blood glucose ([Glu^−^]) and lactate concentrations ([La^−^]) were measured via capillary blood from the earlobe at rest, after the warm‐up and at the end of every incremental step as well as 3‐ and 6 min post exercise (Biosen S‐Line, EKF Diagnostics, Barleben, Germany). Systolic and diastolic blood pressure were also measured at rest, after the warm‐up, every three minutes during and 3‐ and 6 min post exercise. Cardiac response was again continuously measured using a 12‐lead ECG.

Prior to leaving the lab, participants were informed about the testing day inclusion and exclusion criteria for the upcoming trial visits. They were asked to follow a carbohydrate‐rich diet to ensure sufficient pre‐exercise glycogen levels based on the current nutritional recommendations consisting of a minimum of 5 g·kg^−1^·d^−1^ (Kerksick et al. 2018). Participants were also asked to record their complete dietary intake (paper‐based) from two days prior up until the first trial visit (V1) according to the recommendations for carbohydrate intake (≥ 5 g·kg^−1^·d^−1^) and replicate their exact food and liquid intake on the two days before each of the following trial visits (V2–V4).

#### 2.2.2 Trial‐Related Visits and CHO Supplementation (V1‐V4)

Before each trial visit, participants had to refrain from intense exercise for 48 h and remain physically inactive for 24 h. Additionally, the consumption of alcohol was not allowed in the 24 h before the trial visits. To ensure an euhydrated status prior to each trial visit, participants had to demonstrate a urine specific gravity within the range of 1005–1030 mg·dL^−1^ (Oppliger et al. 2005), which was assessed via urine test strips (Combur 10‐Test, Roche Diagnostics, Basel, Switzerland). At the start of each trial visit, participants were screened for the testing day inclusion and exclusion criteria as mentioned above. Again, the IPAQ‐SF was documented to control changes in physical activity levels between trial visits.

Based on the [La^−^] from the CPX test at the screening visit, the first (LTP1) and second lactate turn points (LTP2) were assessed by means of a computer‐aided linear regression break point method (Vienna CPX‐Tool, University of Vienna, Vienna, Austria) integrating the turn point concept. For a more detailed explanation, see (Tschakert and Hofmann 2013; Binder et al. 2008). Participants then performed a constant load cycling exercise until exhaustion at the individual LTP2, which can be interpreted as the equivalent of the anaerobic threshold.

Each cycling test started with a 3 min resting phase on the ergometer, which was followed by a 3 min warm‐up at 20 W before the time to exhaustion (TTE) test began. Here, exercise intensity was defined as the power output at each participant's individual LTP2. Participants were asked to maintain the target workload as long as possible with a cadence between 80 and 100 revolutions per minute (rpm) and motivated by strong verbal encouragement. Exhaustion was defined as cycling below a cadence of 60 rpm for more than three seconds. Participants were allowed to listen to music of their own choice with the instruction that they must also listen to it during the following trial visits.

At each of the four trial visits, participants received one of three different CHO mixtures or a placebo. The CHO mixtures were a combination of glucose (D (+)‐Glucose x H2O 99%, Grüssing GmbH, Filsum, Germany) and fructose (D (−)‐Fructose 99%, Grüssing GmbH, Filsum, Germany) in a 1:1 ratio equalling 20 g·L^−1^·h^−1^, 40 g·L^−1^·h^−1^ or 60 g·L^−1^⋅h^−1^. A combination of glucose and fructose was chosen to ensure the fasting intestinal glucose absorbing effect via two different glucose transporters (GLUT‐5 and SGLT‐1), instead of only targeting SGLT‐1. A noncaloric artificial sweetener (Sucralose, The Hut.com Ltd., Northwich, England) was served as a placebo (0.5 g·L^−1^·h^−1^). The trial visits are subsequently abbreviated as CHO_20_, CHO_40_, CHO_60_ and PLA. To ensure the double‐blind approach, all mixtures were prepared in opaque bottles in advance of each trial visit by a researcher who was not involved in the study procedure.

During the trial visits, CHO ingestion was carried out in the following order: First, during the 3 min sitting phase on the cycle ergometer before the start of the test and then every 10 min during exercise. For the intake of the CHO or PLA solutions during exercise, the spirometric face mask was briefly removed before it was immediately put back on. Participants had to consume each supplement as quickly as possible but at least within 20 s. Since both the amount of CHO (20 g, 40 g or 60 g) and the volume (1 L water) were pre‐determined, participants thus received varying amounts of CHO each dissolved in 167 mL of water at the respective time point dependent on the total amount of CHO or PLA per hour: These amounts were 3.3 g for a total of 20 g·L^−1^·h^−1^, 6.6 g for a total of 40 g·L^−1^·h^−1^ and 10 g for a total of 60 g·L^−1^·h^−1^, respectively. Accordingly, in the PLA trial arm, 0.08 g were consumed at each time point. For better understanding, this means that if a participant had cycled for 25 min, he or she would have consumed the solutions three times (at rest and at minute 10 and 20) equalling a total of 9.9 g CHO in the CHO_20_ trial arm, 19.8 g in the CHO_40_ trial arm and 30 g in the CHO_60_ trial arm, respectively. The amount of placebo consumed is negligible due to the low total amount.

As with the screening visit, a breath‐by‐breath analysis was performed to assess the ventilatory parameters during the constant load cycling exercise. The cardiac response via an ECG as well as systolic and diastolic blood pressure, [Glu^−^], [La^−^] and rating of perceived exertion (RPE) were determined (without the participant having to pause the exercise at any time) at rest, after the warm‐up, every 3 min during exercise, at exhaustion and 3 and 6 min after that.

**FIGURE 1:**
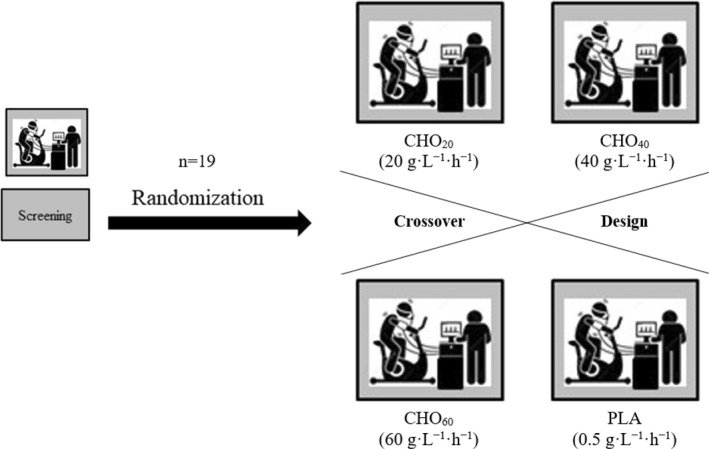
Study flowchart. Carbohydrates (CHO) or placebo (PLA) were given in 10 min intervals dissolved in 167 mL of water equalling a total amount of 20, 40 and 60 g·L^−1^·h^−1^ CHO or 0.5 g·L^−1^·h^−1^. PLA, respectively.

To ensure a standardised testing day protocol, participants performed each individual test at the same time of day on the same day of the week. Any form of food or fluid intake other than the CHO or PLA supplementation were not allowed during the trial visits. To reduce the thermoregulatory variability, participants were asked to wear the same clothing on every trial visit. Between each trial visit, a minimum recovery period of 7 days had to be maintained. A graphical display of the study design can be found below (see Figure 1).

### 2.3 Sample Size Estimation

In advance, a sample size estimation was performed using G × Power (Version 3.1.9.6, University of Kiel, Kiel, Germany) based on a similar study project (Wroble et al. 2018). This analysis resulted in an effect size of *d* = 0.94 (TTE in group 1: 801 ± 58 s and TTE in group 2: 857 ± 61 s). The level of significance was set at 0.05 and the power (1−*β*‐error) at 0.80. This resulted in an a priori calculated sample size of *n* = 19 per trial arm. Since this is a crossover study, the total number of participants required was also 19.

### 2.4 Statistical Analyses

Data were collected and summarised in a Masterfile in Excel (Microsoft Excel, Microsoft Corporation, Redmond, U.S.A.). Once data collection was completed, data were analysed in GraphPad Prism (Version 8.0.2, GraphPad Software Inc., Boston, U.S.A.), tested for normal distribution via the Shapiro–Wilk test and presented according to their distribution including the 95% confidence interval and coefficient of variation. For the primary outcome (TTE), data were compared via a mixed‐effects model with Tukey's multiple comparisons test. A one‐way repeated measures ANOVA with Tukey's multiple comparisons test was conducted to test for significant differences between carbohydrates consumed during the trial arms. A two‐way repeated measures ANOVA or a mixed‐effects analysis was conducted to test for significant differences between the pre‐ and post‐exercise values within and between the respective trial arms. Statistical significance was accepted at *p* < 0.05 (two‐tailed).

## 3 Results

Twenty three healthy, young and moderately active participants were recruited for this study. Four participants had to leave the study prematurely (blood donation, *n* = 1; gastro‐intestinal symptoms, *n* = 1 and injury, *n* = 2). Thus, a total of 19 participants (8 females) were included for statistical analysis. Physical activity measurements assessed before each trial visit were 2429 ± 1319 (CHO_20_), 2910 ± 1799 (CHO_40_), 3598 ± 2131 (CHO_60_) and 2435 ± 1556 (PLA) total metabolic equivalent of task (MET)‐mins∙week^−1^, respectively, and were not statistically different between trial arms (*F* = 1.93 and *p* = 0.13). Participant characteristics, including anthropometrics and body composition, can be found in Table 1.

**TABLE 1:** Participant characteristics including anthropometry and body composition.

	Mean ± SD	95% CI of mean
Age (years)	26.2 ± 1.7	25.5–27.0
Body mass (kg)	70.3 ± 12.0	64.8–75.8
Height (cm)	177.0 ± 9.2	172.7–181.3
Body mass index (kg·m^−2^)	22.4 ± 2.3	21.4–23.5
Total body water (L)	41.7 ± 8.4	37.9–45.5
Fat mass (kg)	14.4 ± 4.9	12.1–16.6
Fat mass (%)	20.6 ± 6.8	17.5–23.7

Abbreviations: CI = confidence interval, SD = standard deviation.

### 3.1 Cardio‐Pulmonary Exercise Test (CPX)

Maximum power output (P_max_) was 3.8 ± 0.5 W·kg^−1^ (268 ± 61 W) and ranged between 3.0 and 4.9 W·kg^−1^ (160 and 380 W). Maximum oxygen uptake (V̇O_2max_), defined as the highest 30 s interval during exercise, was 47.4 ± 7.5 mL·min^−1^ kg^−1^. Maximum [La^−^] was 13.3 ± 1.9 mmol·L^−1^. Respiratory exchange ratio (RER) and heart rate at exhaustion were 1.23 ± 0.07 and 192 ± 14 beats·minute^−1^, respectively. The calculated mean LTP2 was 190 ± 47 W, which equalled 71 ± 3.8% of P_max_, respectively.

### 3.2 Time to Exhaustion (TTE)

**FIGURE 2:**
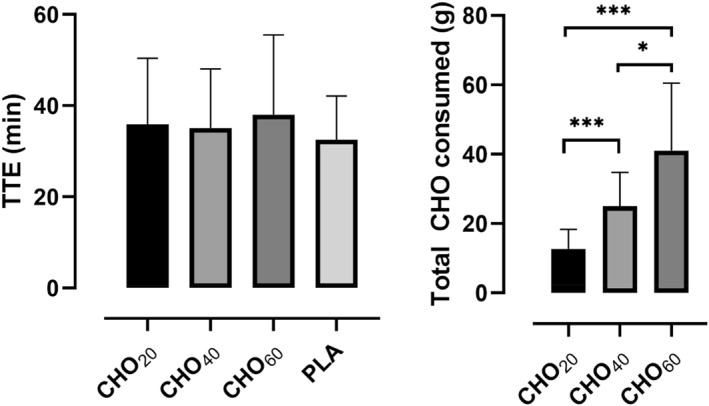
Column bar graph (mean with SD) for the TTE (*p* = 0.11) and carbohydrates consumed (*p* < 0.0001) in comparison of trial arms (the subscript number indicates the amount of CHO consumed per hour for each trial arm). The amount of carbohydrates consumed in the PLA trial arm is negligible and therefore not displayed (***p < 0.0001 and *p < 0.01).

Mean TTE in the different trial arms were 35.9 ± 14.5 min (CHO_20_), 35.1 ± 12.9 min (CHO_40_), 38.0 ± 17.5 min (CHO_60_) and 32.5 ± 9.6 min (PLA), respectively. These times are equivalent to a total of 12.7 ± 5.5 g (CHO_20_), 25.0 ± 9.5 g (CHO_40_) and 41.1 ± 18.9 g (CHO_60_) of carbohydrates consumed (see Figure 1). Coefficients of variation for TTE were 40.4% (CHO_20_), 36.8% (CHO_40_), 46.0% (CHO_60_) and 29.5% (PLA), respectively. A mixed‐effects analysis with Tukey's multiple comparisons test showed that there were no statistically significant differences in TEE between the trial arms (F [2.49, 42.42] = 2.25, *ε* = 0.83 and *p* = 0.11). As for CHO consumed (F [1.45, 26.08] = 33.51, *ε* = 0.72 and *p* < 0.0001), significantly higher amounts of CHO were consumed in the CHO_60_ trial arm (41.0 ± 18.9 g) compared to CHO_20_ (12.7 ± 5.5 g and *p* < 0.0001) and CHO_40_ (25.0 ± 9.5 g and *p* = 0.001, see Figure 2). The amount of carbohydrates consumed in the CHO_40_ trial arm was also significantly higher compared to CHO_20_ (*p* < 0.0001). The multiple comparisons results are displayed in Table 2.

**TABLE 2:** Tukey's multiple comparisons test results for TTE (time to exhaustion) and carbohydrates (CHO) consumed during cycling exercise in comparison of trial arms.

	Mean diff.	95% CI of diff.	Adjusted *p*‐value
TTE (min)			
CHO_20_ versus CHO_40_	0.78	−6.19 to 7.76	0.98
CHO_20_ versus CHO_60_	−2.10	−6.75 to 2.55	0.58
CHO_20_ versus PLA	3.38	−2.58 to 9.34	0.39
CHO_40_ versus CHO_60_	−2.89	−10.25 to 4.48	0.68
CHO_40_ versus PLA	2.59	−4.10 to 9.29	0.69
CHO consumed (g)			
CHO_20_ versus CHO_40_	−12.33	−17.94 to −6.72	< 0.0001***
CHO_20_ versus CHO_60_	−28.37	−38.95 to −17.79	< 0.0001***
CHO_40_ versus CHO_60_	−16.04	−25.67 to −6.42	0.0013*

Abbreviations: CI = confidence interval, Diff. = difference.

### 3.3 Glucose and Lactate

**FIGURE 3:**
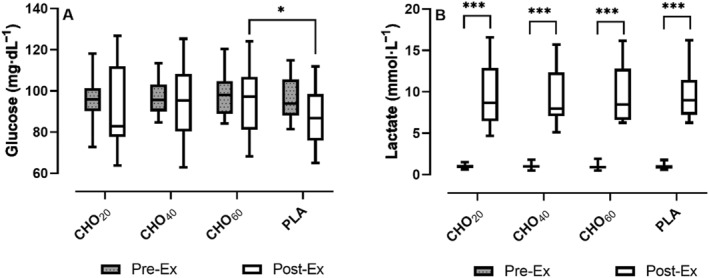
Interleaved box and whiskers plot for glucose (A) and lactate (B) concentrations before (Pre‐ex) and at the end (Post‐ex) of cycling to exhaustion at the LTP2 in comparison of trial arms (***p < 0.0001 and *p < 0.01).

Mean [Glu^−^] at rest (Pre‐ex) was 96.6 ± 10.8 mg·dL^−1^ (CHO_20_), 96.3 ± 8.7 mg·dL^−1^ (CHO_40_), 97.7 ± 9.8 mg·dL^−1^ (CHO_60_) and 96.4 ± 9.9 mg·dL^−1^ (PLA), respectively. A two‐way repeated measures ANOVA with Tukey's multiple comparisons test showed that there were no statistically significant differences between the trial arms under resting conditions (all *p* = 0.99). When compared to the mean [Glu^−^] at the end of exercise (Post‐ex), no statistically significant differences between pre‐ and post‐exercise values were found. Mean post‐exercise [Glu^−^] for CHO_60_ was significantly higher when compared to PLA (95.6 vs. 86.7 mg·dL^−1^ and *p* = 0.03, see Figure 3).

Mean [La^−^] at rest (Pre‐ex) was 1.00 ± 0.27 mmol·L^−1^ (CHO_20_), 0.99 ± 0.32 mmol·L^−1^ (CHO_40_), 0.96 ± 0.37 mmol·L^−1^ (CHO_60_) and 0.95 ± 0.29 mmol·L^−1^ (PLA), respectively. A two‐way repeated measures ANOVA with Tukey's multiple comparisons test showed that there were no statistically significant differences between the trial arms under resting conditions (all *p* = 0.99). Mean [La^−^] at the end of exercise (Post‐ex) was 9.50 ± 3.51 mmol·L^−1^ (CHO_20_), 9.51 ± 3.32 mmol·L^−1^ (CHO_40_), 9.56 ± 3.23 mmol·L^−1^ (CHO_60_) and 9.64 ± 2.99 mmol·L^−1^ (PLA), respectively. When compared to the mean values at exhaustion, [La^−^] was significantly higher at all trial arms (see Figure 3). No significant differences were found between trial arms for the post‐exercise values.

### 3.4 Ventilatory Measurements, Heart Rate and Rating of Perceived Exertion

**FIGURE 4:**
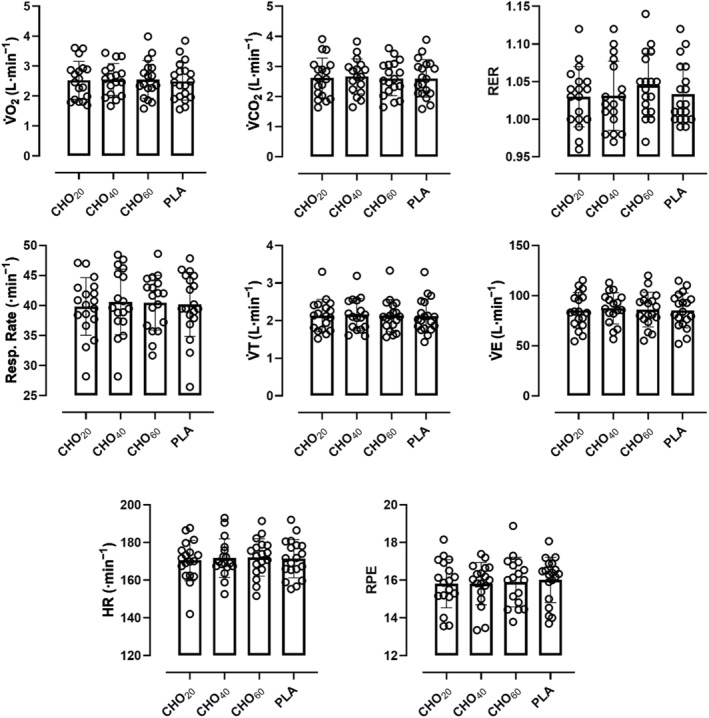
Scatter plot with bar and individual ventilatory, heart rate and RPE values (open circles) in comparison of trial arms.

Mean V̇O_2_ during constant load cycling until exhaustion was 2.52 ± 0.63 L·min^−1^ (CHO_20_), 2.55 ± 0.53 L·min^−1^ (CHO_40_), 2.55 ± 0.63 L·min^−1^ (CHO_60_) and 2.49 ± 0.63 L·min^−1^ (PLA) (F [1.34, 21.91] = 0.18, *ε* = 0.44 and *p* = 0.75). Mean V̇CO_2_ was 2.62 ± 0.66 L·min^−1^ (CHO_20_), 2.67 ± 0.59 L·min^−1^ (CHO_40_), 2.60 ± 0.57 L·min^−1^ (CHO_60_) and 2.61 ± 0.62 L·min^−1^ (PLA) (F [0.94, 15.74] = 0.64, *ε* = 0.31 and *p* = 0.43). Mean RPE was 15.8 ± 1.3 (CHO_20_), 15.8 ± 1.1 (CHO_40_), 15.9 ± 1.3 (CHO_60_) and 16.0 ± 1.2 (PLA) (F [2.57, 42.78] = 0.34, *ε* = 0.86 and *p* = 0.77). Mean HR was 170 ± 11 beats·min^−1^ (CHO_20_), 171 ± 10 beats·min^−1^ (CHO_40_), 172 ± 10 beats·min^−1^ (CHO_60_) and 171 ± 10 beats·min^−1^ (PLA), respectively (F [1.37, 22.75] = 0.23, *ε* = 0.46 and *p* = 0.71, see Figure 4). The multiple comparisons results can be found in the appendix (Appendix A1).

## 4 Discussion

The aim of this double‐blind, randomised and placebo‐controlled crossover trial was to investigate the ergogenic effect of low‐dose CHO supplementations on TTE during a constant load cycling exercise at the LTP2 in healthy moderately active individuals. Our results demonstrated that neither 20, 40 nor 60 g·L^−1^·h^−1^ increased TTE when compared to a placebo. These findings demonstrate that when exercising at high intensities for less than 60 min, exogenous CHO supplementation does not yield a performance enhancing effect if a high carbohydrate diet is followed prior to exercise.

Orally administered CHO supplementation has been the topic of debate for many decades when it comes to enhancing performance outcomes in endurance exercise. Although it has been well established that CHO offers substantial benefits during long‐term glycogen dependant endurance exercise, research regarding its effects during shorter term high‐intensity exercise is contradictory. In this context, our results are in line with the findings of Powers et al., who had 9 endurance‐trained males cycle until fatigue at 85% of their V̇O_2max_. Here, the authors found no performance effect after placebo, water or 7% glucose solution consumption (210 mL every 15 min), totalling 66 g of CHO per hour (Powers et al. 1990). This is further supported by the findings by Bonen et al. and Desbrow et al., who both found no significant performance effect after a CHO supplementation during cycling exercise lasting less than 60 min (Bonen et al. 1981; Desbrow et al. 2004). In the context of this study, total amounts of CHO consumed were 12.7 ± 5.5 g (CHO_20_), 25.0 ± 9.5 g (CHO_40_) and 41.1 ± 18.9 g (CHO_60_), respectively. However, since CHO were given in 10‐minute intervals and the mean TTE of the three CHO trial arms were between 35 and 38 min, it is likely that some of the CHO–especially those administered after 20 and 30 min–were not ready for oxidation due to slow gastric emptying (Murray 1987). Therefore, it might be useful to take the entire amount of CHO at the beginning of exercise to avoid the delay in gastric emptying.

Intriguingly, several studies have reported an increase in performance outcomes. In this context, the effect of CHO mouth washing·has been repeatedly investigated. CHO mouth washing during exercise can stimulate the brain areas of the insula/frontal operculum, orbitofrontal cortex and striatum, which are involved with brain centres responsible for reward and motor control (Chambers et al. 2009). Interestingly, it was shown that if the oral receptors were bypassed via direct infusion of CHO solutions into the venous system, endurance exercise performance was unchanged when compared with a 0.9% saline solution (Carter, Jeukendrup, Mann, et al. 2004). To the best of our knowledge, only one study has also used a TTE design investigating the effects of mouth wash on exercise capacity. Here, Fares and Kayser had 13 healthy nonathletic males who perform several tests on a cycle ergometer. After measuring maximum power output (P_max_), the participants cycled four times at 60% of their P_max_ until exhaustion while rinsing their mouth every five minutes with either a 6.4% maltodextrin solution or water, one time after an overnight fast and another after a high CHO breakfast. Their results demonstrated that mouth rinsing with maltodextrin improved TTE in both pre‐ and post‐prandial states and was accompanied by reductions in the average and maximal RPE whereas HR was unchanged. In our study, we used a 10 min interval with CHO amounts ranging between 3.3 and 10 g per 167 mL of water. Additionally, CHO solutions had to be consumed as quickly as possible. It is possible that these conditions were not sufficient to elicit the performance enhancing effects of a mouthwash protocol. Here, it is usually recommended to ensure an adequate oral CHO exposure (5–10 s every 8–10 min) to provide a sufficient stimulus for the central nervous system (Stellingwerff and Cox 2014).

In general, exercise physiologists proclaim that a person's pre‐exercise glycogen levels are the single most important acutely modifiable determinant of endurance capacity. However, in a recent review, Noakes stated that in most trials not only were muscle glycogen concentrations low at exhaustion but hypoglycaemia was also always present (Noakes 2022). Low blood glucose concentrations would reflect low liver rather than low muscle glycogen stores, eventually leading to fatigue and an impairment of performance. In our study, although post‐exercise [Glu^−^] for CHO_60_ were significantly higher when compared to PLA, [Glu^−^] at exhaustion were well above the hypoglycaemic range in all trial arms. Most importantly, [Glu^−^] did not change between pre‐ and post‐exercise conditions in all trial arms, even though the amounts of total CHO consumed during exercise differed by up to three times between the CHO trial arms (CHO_20_: 12.7 g, CHO_40_: 25.0 g and CHO_60_: 41.0 g). It has been demonstrated that despite diverse types and amounts of CHO consumed before and during exercise, [Glu^−^] remained stable and did not change until the end of exercise (Podlogar, Cirnski, et al. 2022). This was partly explained by higher glucose oxidation rates with larger exogenous CHO amounts consumed. Since we explicitly used low amounts of CHO in this study, we would argue that this was also the reason for the unchanged [Glu^−^] in the three CHO trial arms. It is also reasonable to assume that low liver glycogen levels are not responsible for the onset of fatigue during cycling at the LTP2. In this context, it is worth mentioning that the participants in this study were following the current guidelines for CHO intake for athletes involved in moderate amounts of intense training (e.g., 2–3 h per day of intense exercise performed 5–6 times per week) to ensure adequate muscle glycogen loading (Kerksick et al. 2018). Exercise‐induced hypoglycaemia defined as [Glu^−^] < 70 mg·dL^−1^ and as postulated previously (Noakes 2022) was not present in any of the trial arms and is therefore not associated with the development of the fatigue process in this study.

In contrast, previous studies have documented significant changes in [Glu^−^] especially when following low CHO diets prior to exercise (Christensen and Hansen 1939). The body's own glycogen stores can provide enough energy to master more severe exercise intensities, for example, at the LTP2, especially when glycogen stores are adequately loaded. This view is further supported by the fact that previous studies have demonstrated that exercising at comparable intensities was impaired when glycogen stores were empty pre‐exercise and aligns with the general rationale, that during short duration (< 1 h), high‐intensity exercise situations the type and/or amount of CHO and its ability to be absorbed and sized appear irrelevant to enhancing performance (Stellingwerff and Cox 2014; Carter, Jeukendrup, Mann, et al. 2004).

On a methodological basis, this study has several strengths and limitations. First, the double‐blind, randomised and placebo‐controlled crossover trial design of our study aimed to address existing gaps in the literature by systematically evaluating the impact of low‐dose CHO supplementation during constant load cycling at the LTP2. This design involved each participant experiencing all trial arms in a random order across multiple exercise sessions, effectively controlling for inter‐individual variability and allowing for within‐subject comparisons. The double‐blinding of both participants and investigators to the assigned supplementations enhances the reliability of the results, as it prevents conscious or subconscious biases from influencing the study's outcome. Moreover, the crossover nature of the trial design optimises statistical power by enabling the use of each participant as their control, thereby reducing the potential impact of confounding variables. It is our opinion that this methodological strength ensures a robust examination of the dose–response relationship between carbohydrate supplementation and constant load cycling performance, providing valuable insights for both researchers and practitioners. As the demand for evidence‐based practices continues to grow, our research provides actionable insights that may benefit researchers, athletes and coaches in optimising performance strategies.

In contrast, there are several limitations to this study. First, we did not carry out a familiarisation visit for the TTE protocol. Although we did not see any differences in the trial arms regarding physiological or subjective markers, future studies may incorporate a familiarisation visit, especially when recruiting participants with no experience in exercise‐induced fatigue. Second, it has been repeatedly demonstrated that the menstrual cycle can affect exercise performance (McNulty et al. 2020). Since we did not schedule the trial visits for our female participants according to the respective phases, we cannot tell whether their performance was possibly affected by the menstruation cycle. Furthermore, we controlled for hydration status via a urine specific gravity test, although the chosen range (1005–1030 mg·dL^−1^) might have been too wide to accurately detect dehydration‐related changes. Therefore, we cannot ultimately say whether performance outcomes may have been influenced by differences in hydration status. In any case, the request to exactly replicate the diet and thus also the fluid intake prior to the trial visits should have contributed to the fact that the hydration status is likely be comparable. Our dietary CHO recommendation for the period prior to the study visits was based on the upper range of the current guidelines for maintaining glycogen stores. However, since we did not directly measure intramuscular or hepatic glycogen stores, we cannot tell whether the dietary recommendations truly led to sufficiently filled substrate stores. Lastly, it must be mentioned that our sample size calculation was based on a study that used only two trial arms with a SD of about 7% for each trial arm. Given that the calculated SDs for each CHO trial arm in our study were substantially higher, it is likely that the results presented here are underpowered. Therefore, future studies should incorporate lager samples with a higher statistical power.

## 5 Conclusion

Consuming low amounts of exogenous CHO in the form of a glucose–fructose combination (1:1) does not improve TTE during cycling at the LTP2 in healthy moderately active individuals when compared to a PLA. Therefore, if a carbohydrate‐rich diet is followed several days prior to exercise, oral carbohydrate administration does not offer any performance‐enhancing effect on time to exhaustion when cycling at high intensities such as the LTP2.

## Ethics Statement

The local ethics committee of the University of Bayreuth (Germany) approved the study protocol (O 1305/1 ‐ GB, 2 November 2022), and the study was conducted in conformity with the declaration of Helsinki and guidelines for good clinical practice. Additionally, the study protocol was registered at the German Clinical Trial Register (DRKS‐ID: DRKS00030531). Before any trial related activities, potential participants were informed about the study protocol and participants gave their written informed consent.

## Conflicts of Interest

The authors declare no conflicts of interest.

## Appendix

**TABLE A1:** Tukey's multiple comparisons test results for ventilatory, heart rate and rating of perceived exertion values in comparison of trial arms.

		Mean diff.	95% CI of diff.	Adjusted *p*‐value
V̇O_2_	CHO_20_ versus CHO_40_	−0.02	−0.12 to 0.07	0.87
	CHO_20_ versus CHO_60_	−0.03	−0.16 to 0.09	0.89
	CHO_20_ versus PLA	0.03	−0.07 to 0.13	0.83
	CHO_40_ versus CHO_60_	−0.01	−0.15 to 0.13	0.99
	CHO_40_ versus PLA	0.06	−0.08 to 0.19	0.68
V̇CO_2_	CHO_20_ versus CHO_40_	−0.05	−0.13 to 0.04	0.39
	CHO_20_ versus CHO_60_	0.02	−0.10 to 0.13	0.97
	CHO_20_ versus PLA	0.02	−0.07 to 0.10	0.95
	CHO_40_ versus CHO_60_	0.07	−0.03 to 0.16	0.23
	CHO_40_ versus PLA	0.06	−0.03 to 0.16	0.27
RER	CHO_20_ versus CHO_40_	−0.01	−0.03 to 0.03	0.99
	CHO_20_ versus CHO_60_	−0.02	−0.05 to 0.02	0.58
	CHO_20_ versus PLA	−0.01	−0.03 to 0.019	0.98
	CHO_40_ versus CHO_60_	−0.01	−0.05 to 0.02	0.66
	CHO_40_ versus PLA	−0.01	−0.03 to 0.03	0.99
Resp. Rate	CHO_20_ versus CHO_40_	−0.77	−2.78 to 1.23	0.69
	CHO_20_ versus CHO_60_	−0.63	−2.71 to 1.45	0.82
	CHO_20_ versus PLA	−0.35	−2.03 to 1.34	0.93
	CHO_40_ versus CHO_60_	0.14	−2.01 to 2.29	0.99
	CHO_40_ versus PLA	0.43	−0.90 to 1.76	0.80
V̇T	CHO_20_ versus CHO_40_	−0.02	−0.09 to 0.04	0.75
	CHO_20_ versus CHO_60_	0.01	−0.07 to 0.07	0.99
	CHO_20_ versus PLA	0.01	−0.06 to 0.09	0.94
	CHO_40_ versus CHO_60_	0.03	−0.04 to 0.09	0.65
	CHO_40_ versus PLA	0.04	−0.04 to 0.12	0.56
V̇E	CHO_20_ versus CHO_40_	−1.91	−5.80 to 2.00	0.51
	CHO_20_ versus CHO_60_	−0.74	−5.37 to 3.89	0.97
	CHO_20_ versus PLA	0.21	−3.21 to 3.62	0.99
	CHO_40_ versus CHO_60_	1.17	−3.12 to 5.47	0.86
	CHO_40_ versus PLA	2.12	−1.93 to 6.17	0.46
HR	CHO_20_ versus CHO_40_	−1.25	−4.87 to 2.37	0.75
	CHO_20_ versus CHO_60_	−1.64	−8.21 to 4.93	0.89
	CHO_20_ versus PLA	−0.91	−3.90 to 2.09	0.82
	CHO_40_ versus CHO_60_	−0.39	−3.86 to 3.09	0.98
	CHO_40_ versus PLA	0.34	−2.82 to 3.51	0.99
RPE	CHO_20_ versus CHO_40_	−0.01	−0.79 to 0.78	> 0.99
	CHO_20_ versus CHO_60_	−0.09	−0.78 to 0.59	0.98
	CHO_20_ versus PLA	−0.20	−0.78 to 0.39	0.78
	CHO_40_ versus CHO_60_	−0.08	−0.81 to 0.64	0.99
	CHO_40_ versus PLA	−0.19	−0.80 to 0.41	0.80
